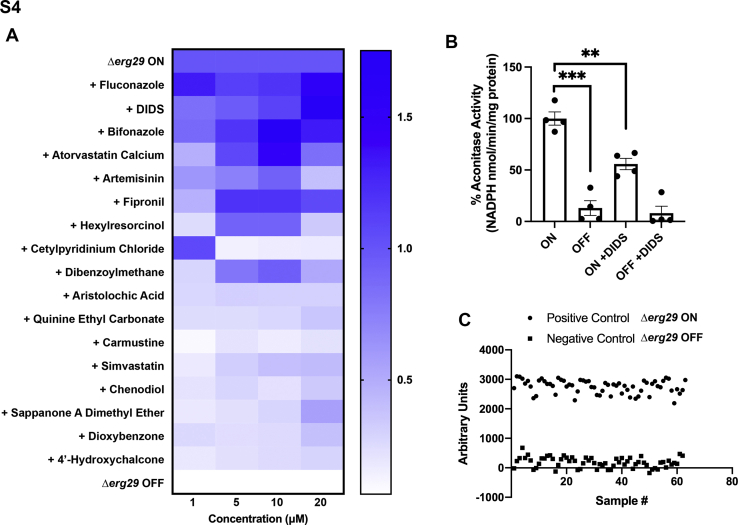# Posttranslational regulation of mitochondrial frataxin and identification of compounds that increase frataxin levels in Friedreich’s ataxia

**DOI:** 10.1016/j.jbc.2022.101982

**Published:** 2022-04-25

**Authors:** Peter T. Hackett, Xuan Jia, Liangtao Li, Diane M. Ward

**Affiliations:** Department of Pathology, Division of Microbiology and Immunology, University of Utah School of Medicine, Salt Lake City, Utah, USA

**Keywords:** compounds, frataxin, iron, iron–sulfur clusters, mitochondria, ROS, screen, yeast, Yfh1, 4′-OHC, 4′- hydroxychalcone, CDDO, 2-cyano-3,12-dioxoolean-1,9-dien-28-oic acid 9, CPCL, cetylpyridinium chloride, DBM, dibenzoylmethane, DIDS, 4,4-diisothiocyanostilbene-2,2-sulfonic acid, FDA, Food and Drug Administration, FeNTA, Fe-nitrotriacetic acid, FRDA, Friedreich’s ataxia, Fxn, frataxin, Ig, immunoglobulin, ISC, Fe–S cluster, Nrf2, nuclear factor erythroid 2–related factor 2, ROS, reactive oxygen species, RT-qPCR, reverse transcription quantitative PCR

## Abstract

Friedreich’s ataxia (FRDA) is a degenerative disease caused by a decrease in the mitochondrial protein frataxin (Fxn), which is involved in iron–sulfur cluster (ISC) synthesis. Diminutions in Fxn result in decreased ISC synthesis, increased mitochondrial iron accumulation, and impaired mitochondrial function. Here, we show that conditions that result in increased mitochondrial reactive oxygen species in yeast or mammalian cell culture give rise to increased turnover of Fxn but not of other ISC synthesis proteins. We demonstrate that the mitochondrial Lon protease is involved in Fxn degradation and that iron export through the mitochondrial metal transporter Mmt1 protects yeast Fxn from degradation. We also determined that when FRDA fibroblasts were grown in media containing elevated iron, mitochondrial reactive oxygen species increased and Fxn decreased compared to WT fibroblasts. Furthermore, we screened a library of FDA-approved compounds and identified 38 compounds that increased yeast Fxn levels, including the azole bifonazole, antiparasitic fipronil, antitumor compound dibenzoylmethane, antihypertensive 4-hydroxychalcone, and a nonspecific anion channel inhibitor 4,4-diisothiocyanostilbene-2,2-sulfonic acid. We show that top hits 4-hydroxychalcone and dibenzoylmethane increased mRNA levels of transcription factor nuclear factor erythroid 2–related factor 2 in FRDA patient-derived fibroblasts, as well as downstream antioxidant targets thioredoxin, glutathione reductase, and superoxide dismutase 2. Taken together, these findings reveal that FRDA progression may be in part due to oxidant-mediated decreases in Fxn and that some approved compounds may be effective in increasing mitochondrial Fxn in FRDA, delaying disease progression.

The ability to synthesize Fe–S clusters (ISCs) is an essential process for all eukaryotes and most prokaryotes. ISCs act as prosthetic groups in many proteins including enzymes such as aconitase, DNA repair enzymes, and electron transport proteins (for review see (1)). Defects in mitochondrial ISC synthesis result in human diseases including Friedreich’s ataxia (FRDA), sideroblastic anemia with ataxia, and myopathy with ISCU deficiency (for review see (2, 3)). FRDA is a progressive disease that affects multiple tissues with the most pronounced phenotypes in neuromuscular and cardiac tissue (4, 5, 6, 7). The mutated gene responsible for FRDA is *FXN*, which encodes for the highly conserved mitochondria ISC protein frataxin (Fxn) (8, 9). FRDA patients have markedly reduced levels of Fxn, which result in diminished ISC synthesis, increased mitochondrial iron, and elevated mitochondrial reactive oxygen species (ROS) and biochemical changes that have been shown in both cardiac tissue of FRDA patients and FRDA animal models (10, 11, 12, 13, 14). Mature Fxn in the mitochondrial matrix has been shown to interact with ISC assembly enzyme Iscu and the cysteine desulfurase Nfs1 to increase the activity of Nfs1 (15, 16, 17).

In FRDA, most patients have reduced levels of Fxn due to an intron expansion that results in reduced transcripts (18, 19). However, a wide range of missense and nonsense mutations affecting Fxn levels have also been reported (20, 21, 22). The severity of the disease is directly correlated with the levels of mature mitochondrial Fxn. Recent studies have shown decreased Fxn levels can result from causes other than mutations in the *FXN* gene. Doxorubicin treatment of mammalian cardiac cells has been shown to result in decreased Fxn levels due to increased Fxn degradation (23, 24). More recently, studies in yeast have shown that mutations in *ERG29* lead to increased levels of 4′methyl sterol intermediates leading to an iron-dependent oxidation of mitochondrial Yfh1 (the yeast Fxn homolog) resulting in decreased Yfh1 levels, decreased ISC production, and impaired viability and cell growth (25, 26). Many studies have implicated elevated levels of ROS in FRDA pathology (27, 28). Here, we show that increased mitochondrial ROS resulted in a decrease in mitochondrial Yfh1 half-life due to degradation by the mitochondrial Lon protease Pim1. We also showed that increasing mitochondrial iron export through overexpression of the mitochondrial iron exporter *MMT1* (29, 30) preserved Yfh1 levels even in the absence of Erg29. We confirmed that increased mitochondrial ROS gave rise to decreased mammalian Fxn while partner proteins Iscu and Nfs1 levels were unchanged in the H9C2 cardiomyocyte doxorubicin model. We demonstrated that FRDA patient fibroblasts were more susceptible to iron-dependent Fxn degradation and that inhibiting the Lon protease protected mammalian mitochondrial Fxn from ROS-induced degradation. We screened a library of the Food and Drug Administration (FDA)-approved compounds for their ability to increase yeast Yfh1 levels when *ERG29* expression was shut off and identified compounds that increased Yfh1 levels and cell growth. Compounds that showed the highest efficacy in yeast also showed increased mature mitochondrial Fxn levels in doxorubicin-treated cardiomyocytes and FRDA fibroblasts. Our data provide a possible explanation for the progression of disease in FRDA due to further loss of Fxn, and we identify compounds that may be efficacious in slowing the progression of FRDA.

## Results

### Yfh1 half-life is decreased and mitochondrial ROS appear rapidly after loss of *ERG29* expression

**Figure 1:**
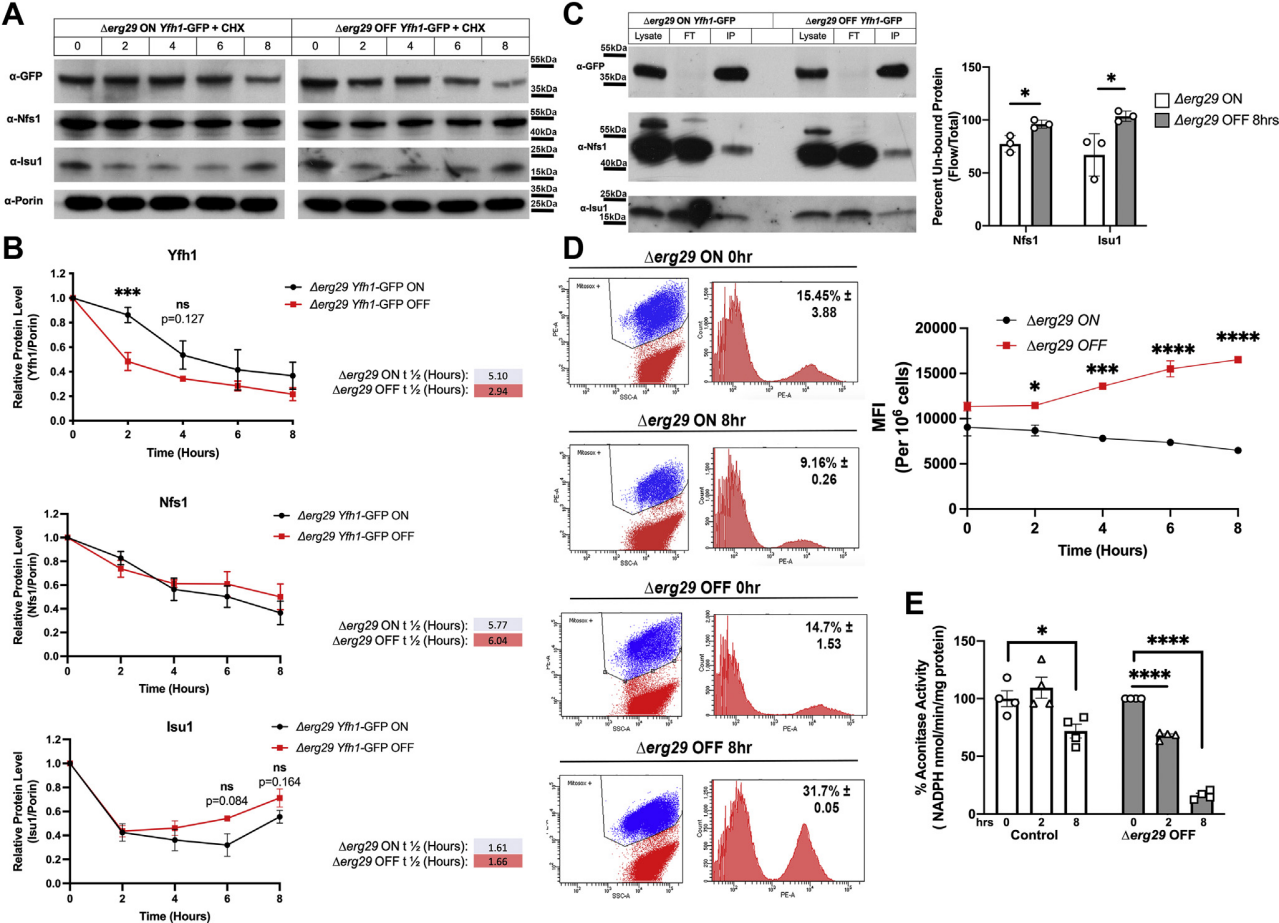
**Loss of Erg29 results in a reduced half-life for Yfh1.***A*, *Δerg29pGAL1ERG29YFH1-GFP* cells were grown overnight in galactose (ON) (*black*) and then shifted to glucose (OFF) (*red*) in the presence of 100 μM cycloheximide for 0 to 8 h. Mitochondria were isolated and Yfh1-GFP, Nfs1, Isu1, and Porin levels determined by Western blot. Porin levels were used as a loading control. A time course of representative blots for Yfh1-GFP, Nfs1, Isu1, and Porin from the same experiment are shown. n = 3. *B*, Western blots from three biologic replicate experiments were quantified and graphed as described in Experimental procedures. Half-lives were determined for Yfh1-GFP, Nfs1, and Isu1 as described in Experimental procedures using one-phase decay. *C*, Yfh1-GFP was immunopurified from samples as in (*A*) and Yfh1-GFP, Nfs1, and Isu1 levels in the immunopurification determined. A representative blot is shown. The data are graphed as the percent remaining in the flow through. Eight percent of the total lysate was loaded (40 μl out of 500 μl total) for anti-GFP blots, while anti-Nfs1 and anti-Isu1 blots were loaded with 0.2% of the total lysate. For IP, 20% of the total IP was loaded. n = 3 replicate experiments. *D*, mitochondrial ROS were measured in cells as in (*A*) using MitoSOX as described (26). A representative flow plot is shown for the 0 and 8 h time points. The mean fluorescence intensity (MFI) was determined over time of *ERG29* shut off compared to *ERG29* ON cells. n = 3 replicate experiments. *E*, aconitase levels were determined as described previously (26) from WT (control) and *Δerg29pGAL1ERG29YFH1-GFP* cells grown in galactose and shifted to glucose (OFF) for 0, 2, or 8 h. Error bars in all panels represent SEM (n ≥ 3 biologic replicate experiments). FT, flow through; IP, immunopurification; L, total lysate; ROS, reaction oxygen species.

**Supplemental Figure 1:**
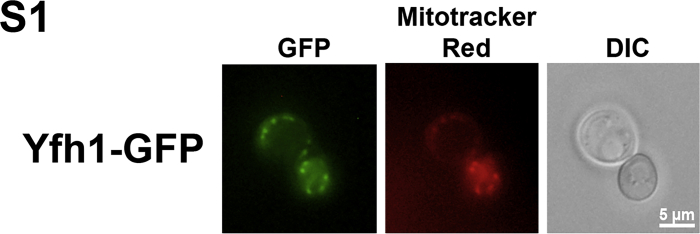


Previously, we reported that loss of Erg29, a protein involved in ergosterol synthesis in yeast, resulted in mitochondrial dysfunction and decreases in the ISC synthesis protein Yfh1 (26). We reported that the decrease in Yfh1 protein levels was not due to a change in *YFH1* transcripts (26), suggesting that the change in Yfh1 levels was due to posttranslational regulation. To determine if the half-life of mitochondrial Yfh1 was altered, we generated a *Δerg29* yeast strain containing a plasmid with expression of *ERG29* regulated by the *GAL1* promoter. In the presence of galactose, cells express *ERG29* (ON) and when grown in glucose as a carbon source, *ERG29* is not expressed (OFF). The strain also contains the coding sequence of GFP cloned in frame into the carboxyl terminus of the endogenous locus of *YFH1*. We refer to this strain as *ERG29 YFH1-GFP* shut off cells. Yfh1-GFP was found to localize to the mitochondria as previously reported (Fig. S1) (25). We grew these cells in galactose (ON) and then shifted them to glucose (OFF) in the presence of cycloheximide to block new translation. We observed a significant diminution in Yfh1-GFP levels in *ERG29* OFF cells compared to *ERG29* ON cells (Fig. 1*A*). Quantification of Western blots revealed that the half-life of Yfh1-GFP in *ERG29* ON cells (5.10 h) was similar to that previously reported for Yfh1 (4.9 h) (31) (Fig. 1*B*). In *ERG29* OFF cells, we observed a rapid decrease in the half-life of mitochondrial Yfh1-GFP (2.94 h), whereas the half-lives of Nfs1 and Isu1, other ISC synthesis proteins that act in the early ISC assembly complex (32, 33), were unaltered. The amount of Nfs1 and Isu1 found complexed to Yfh1-GFP in control cells (*ERG29* ON) was about 20 to 30%, with 70 to 80% unbound at any given time (Fig. 1*C*). As expected, the levels of unbound Nfs1 and Isu1 increased in the *ERG29* shut off cells. The change in Yfh1 half-life was observed almost immediately after turn off of *ERG29* expression, suggesting that there is either a signal or modification on Yfh1 that mediates increased turnover. The decreases in Yfh1 levels seen as early as 2 h after *ERG29* shut off resulted in increased mitochondrial ROS as measured by the MitoSOX mean fluorescence intensity (Fig. 1*D*) and diminished ISC synthesis as measured by aconitase activity (Fig. 1*E*). This result suggests that the levels of Yfh1 can be “rate-limiting” for ISC synthesis. We also noted that control cells grown in galactose showed reduced aconitase activity suggesting that ISC synthesis may not meet the demands required by growth in galactose.

### The Lon protease Pim1 is responsible for increased turnover of Yfh1 under mitochondrial oxidative stress

**Figure 2:**
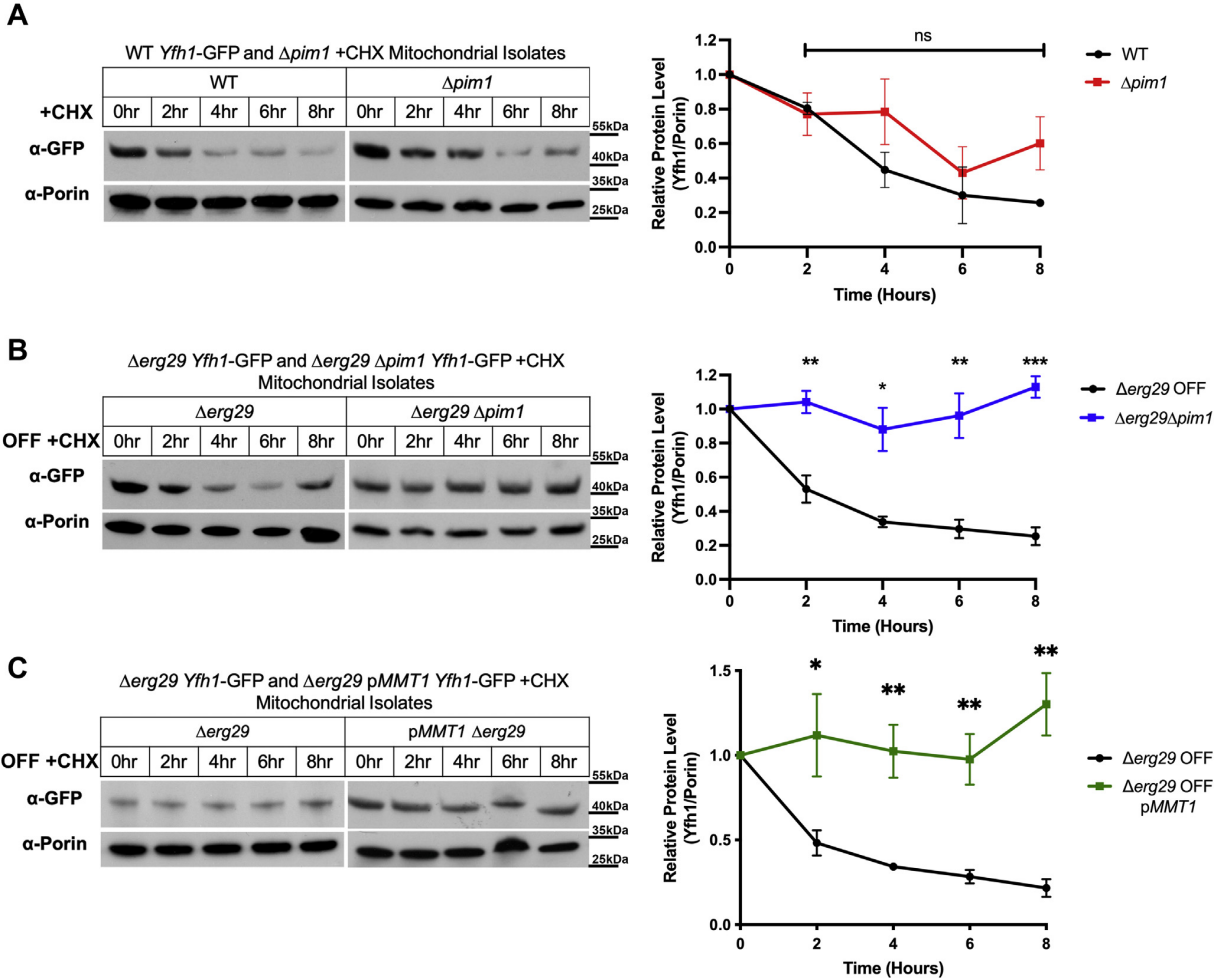
**Pim1 is responsible for mitochondrial Yfh1 degradation in *ERG29* shut off cells.***A*, WT (*black*) and *Δpim1* (*red*) cell containing genomically tagged *YFH1-GFP* were grown in galactose and shifted to glucose in the presence of 100 μM cycloheximide for time, mitochondria isolated, and Yfh1-GFP and Porin levels determined by Western blot as in Figure 1. Representative blots are shown. Quantification was done as described in Experimental procedures. n = 3 replicate experiments. *B*, levels of Yfh1-GFP and Porin were determined in *Δerg29pGAL1ERG29* (*black*) and *Δerg29pGAL1ERG29Δpim1* (*blue*) containing genomically tagged *YFH1-GFP* grown in glucose ± 50 nM β-estradiol as previously described (26). The removal of β-estradiol mimics galactose to glucose shut off of ERG29. This was done in the presence of 100 μM cycloheximide for time as in (*A*). *C*, levels of Yfh1-GFP and Porin were determined in *Δerg29pGAL1ERG29* (*black*) and *Δerg29pGAL1ERG29pMMT1* (*green*) grown as in (*A*). Representative Western blots are shown for each. All blots were quantified using Fiji ImageJ software and the changes in Yfh1-GFP determined over time using Porin as a control. Error bars in all panels represent SEM (n ≥ 3 replicate experiments). ROS, reaction oxygen species.

Previous studies suggest that immature Fxn levels can be changed by inhibiting specific protease pathways in mammalian FRDA cells including the proteasome and autophagy pathways along with inhibiting the mitochondrial protease PITRM1 (34, 35, 36, 37). Mitochondria possess several different protease systems to maintain mitochondrial homeostasis and cellular health (38, 39, 40, 41, 42). We focused on the Lon protease Pim1, a yeast mitochondrial protease known to mediate oxidized protein turnover (43, 44). To determine if Pim1 is responsible for increased Yfh1 turnover in *ERG29 OFF* cells, we generated strains expressing *YFH1-GFP* with a deletion of *PIM1* in WT and *Δerg29pGAL1ERG29* cells. Loss of Pim1 resulted in a slight increase in Yfh1-GFP half-life in WT mitochondria, although not to significance (Fig. 2*A*), whereas, deletion of *PIM1* in *Δerg29pGAL1ERG29* shut off cells resulted in a dramatic increase in the half-life of mitochondrial localized Yfh1-GFP with minimal Yfh1-GFP degradation observed when *ERG29* was shut off (Fig. 2*B*). In addition, overexpression of the mitochondrial iron exporter Mmt1 protected Yfh1 from Pim1-mediated degradation in *ERG29* OFF conditions (Fig. 2*C*). These results suggest that mitochondrial iron accumulation and increased mitochondrial oxidant-mediated targeting of Yfh1 are responsible for increased Yfh1 degradation through Pim1.

### Mammalian Fxn levels are reduced in doxorubicin-treated cardiomyocytes and iron-loaded FRDA fibroblasts

**Figure 3:**
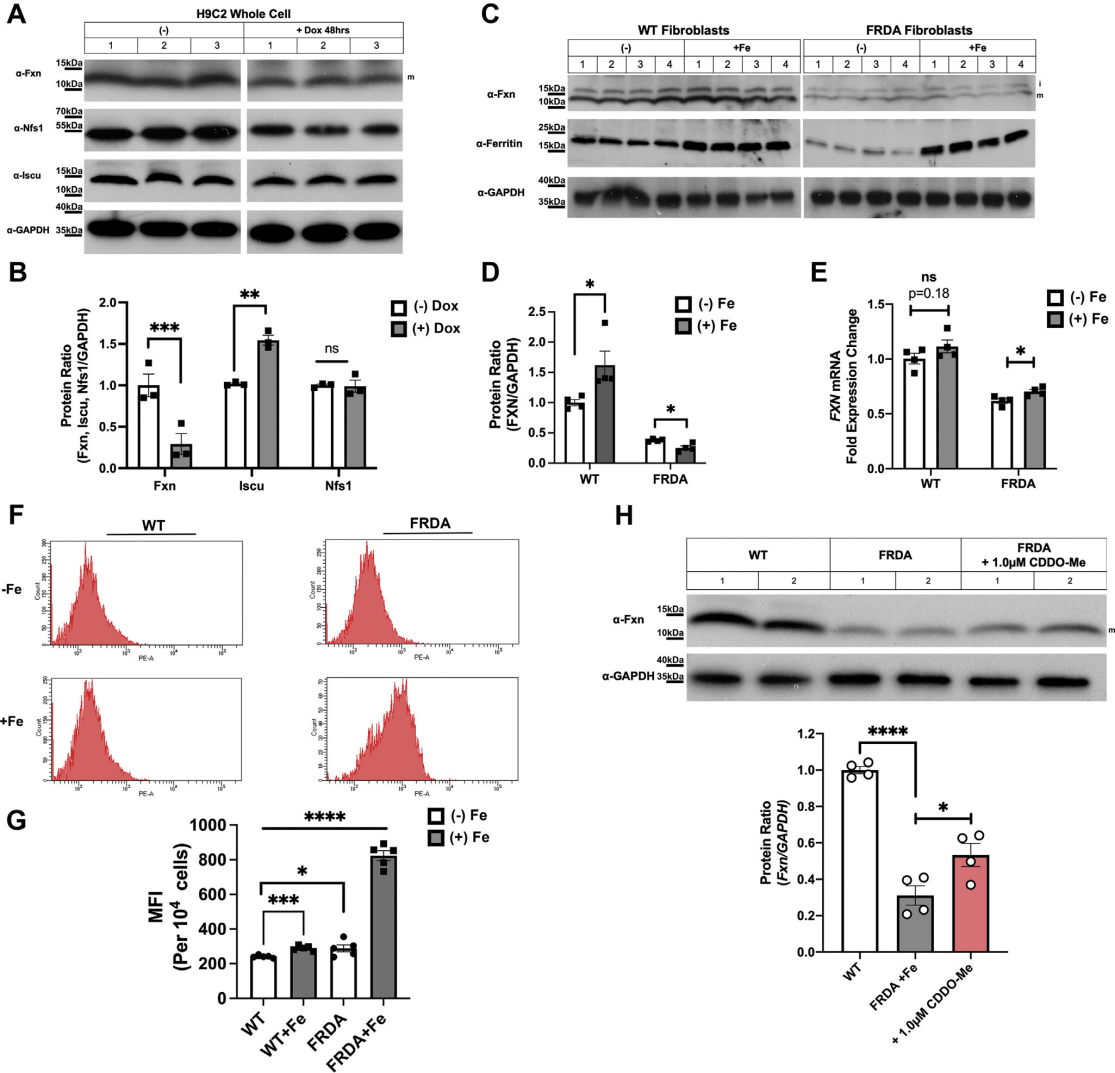
**Frataxin levels are reduced in doxorubicin exposed cardiomyocytes and FRDA fibroblast exposed to increased iron.***A*, rat H9C2 cells were incubated with or without 10 μM doxorubicin for 48 h, cells were harvested, washed in PBS, lysed in 1% Triton X-100, 150 mM NaCl, 10 mM Tris–HCl, 0.5 mM Na_4_EDTA pH 7.2, 0 °C 30 min, and centrifuged for 30 min at 14,000*g*. Supernatants were boiled in SDS-PAGE loading buffer, run on 4 to 20 SDS-PAGE and Fxn, Nfs1, Iscu, and GAPDH levels determined by Western blot. A representative blot with three replicates is shown. n = 3 replicate experiments. *B*, blots from (*A*) were quantified using GAPDH as a loading control and data normalized to 1.0 using samples without doxorubicin as a control. Error bars represent SEM (n = 3 replicate experiments). *C*, WT and FRDA fibroblasts were grown in the absence or presence of 20 μM FeNTA for 48 h, harvested, and lysed as described in (*A*) and Fxn, ferritin, and GAPDH levels determined by Western blot. Representative blots with four experimental replicates are shown. n = 4 replicate experiments. *D*, Western blots from (*C*) were quantified as described in Experimental procedures and data normalized to 1.0 using WT samples without FeNTA as the control. Error bars represent SEM (n = 4 replicate experiments). *E*, *FXN* and *ACTB* transcript levels from cells as in (*C*) were quantified using RT-qPCR as described in Experimental procedures. (n = 4 replicate experiments with 3–4 technical replicates/experiment). *F*, mitochondrial ROS were determined using MitoSOX in FRDA cells as in (*C*) with an example graph shown for WT and FRDA ± Fe. *G*, MitoSOX values from five biologic replicates as in (*F*) were graphed and expressed as the MFI. *H*, cells grown in supplemental FeNTA as in (*C*) were treated with the Lon1 protease inhibitor CDDO-Me (1.0 μM) for 24 h and Fxn and GAPDH levels determined and quantified n = 4 replicate experiments. CDDO, 2-cyano-3,12-dioxoolean-1,9-dien-28-oic acid 9; FeNTA, Fe-nitrotriacetic acid; FRDA, Friedreich’s ataxia; Fxn, frataxin; ROS, reactive oxygen species; RT-qPCR, reverse transcription quantitative PCR.

Previous studies have shown doxorubicin treatment of cardiomyocytes results in increased mitochondrial ROS, decreased Fxn levels, and decreased mitochondrial function (23, 45, 46). Overexpression of Fxn protects against oxidant-mediated cell death. We examined if other ISC synthesis proteins were affected by doxorubicin exposure. When the rat cardiomyocyte cell line H9C2 was treated with doxorubicin, there was a marked decrease in Fxn levels, whereas, levels of the ISC scaffolding protein Iscu increased and Nfs1 levels were unaltered (Fig. 3, *A* and *B*). (Quantification of Western blots was done on mature Fxn). This suggests that the increased mitochondrial ROS in doxorubicin-treated cardiomyocytes specifically affect Fxn levels, similar to what we observed in our yeast *ERG29* shut off model (Figs. 1 and 2).

It is important to note that decreases in Fxn levels below 50% have been shown to result in mitochondrial iron accumulation in yeast cells and in mammalian models of ISC defects (25, 47, 48). We hypothesized that increasing iron levels in the mitochondrial of FRDA patients would further reduce mitochondrial Fxn levels. To our knowledge, the levels of mitochondrial Fxn over time of concurrent mitochondrial iron accumulation in patients have not been evaluated. To test this hypothesis, we grew WT and FRDA fibroblasts in 20 μM Fe-nitrotriacetic acid (FeNTA) and measured Fxn levels and mitochondrial ROS. Fxn levels were increased in WT cells grown in the presence of FeNTA (Fig. 3, *C* and *D*). In contrast, Fxn levels were significantly reduced in FRDA cells under these same conditions. Both WT and FRDA showed increased levels of ferritin, indicating that both WT and FRDA cells were iron loaded. To determine if the changes in Fxn levels were due to changes in transcripts, we quantified *FXN* mRNA levels using reverse transcription quantitative PCR (RT-qPCR). We observed a small but significant increased *FXN* transcripts in FeNTA grown FRDA cells (Fig. 3*E*). These results show that the decrease in Fxn protein in FeNTA-exposed FRDA cells was not due to a decrease in *FXN* transcripts. The increase in cellular iron levels slightly increased mitochondrial ROS in WT cells and dramatically increased mitochondrial ROS in FRDA cells (Fig. 3, *F* and *G*). Our yeast studies showed that when mitochondrial ROS were increased due to iron accumulation, yeast Fxn (Yfh1) was degraded by the Lon protease Pim1 (Fig. 2), which is known to target oxidized proteins (49). To determine if mammalian Fxn is a target of the Lon1 protease, we utilized a Lon1 protease inhibitor 2-cyano-3,12-dioxooleana-1,9-dien-28-oic acid methyl ester (CDDO)–Me to prevent degradation of oxidized mitochondrial proteins (50). We confirmed that the CDDO-Me acted as a Lon inhibitor as the levels of a known Lon target, aconitase 2 (51, 52), were increased when FRDA cells were treated with CDDO-Me (Fig. S3). When FRDA cells grown in FeNTA were treated with CDDO-me, we observed a significant increase in Fxn levels (Fig. 3*H*). These results support a role for the Lon protease in mitochondrial iron/oxidant-mediated decreased Fxn levels similar to that observed in yeast (Fig. 2) and for other yeast ISC proteins (53). As expected, the levels of Fxn did not approach that of WT fibroblasts as the genetic mutation harbored in FRDA patient cells already shows a >50% decrease in Fxn due to reduced *FXN* mRNA levels. Fxn levels did return to pre iron exposure levels. We noted that extended incubations (>24 h) with 1 μM CDDO-Me were toxic to cells (data not shown) not allowing for extended time course evaluations of Fxn turnover.

### Screen for compounds that increase Fxn levels

**Figure 4:**
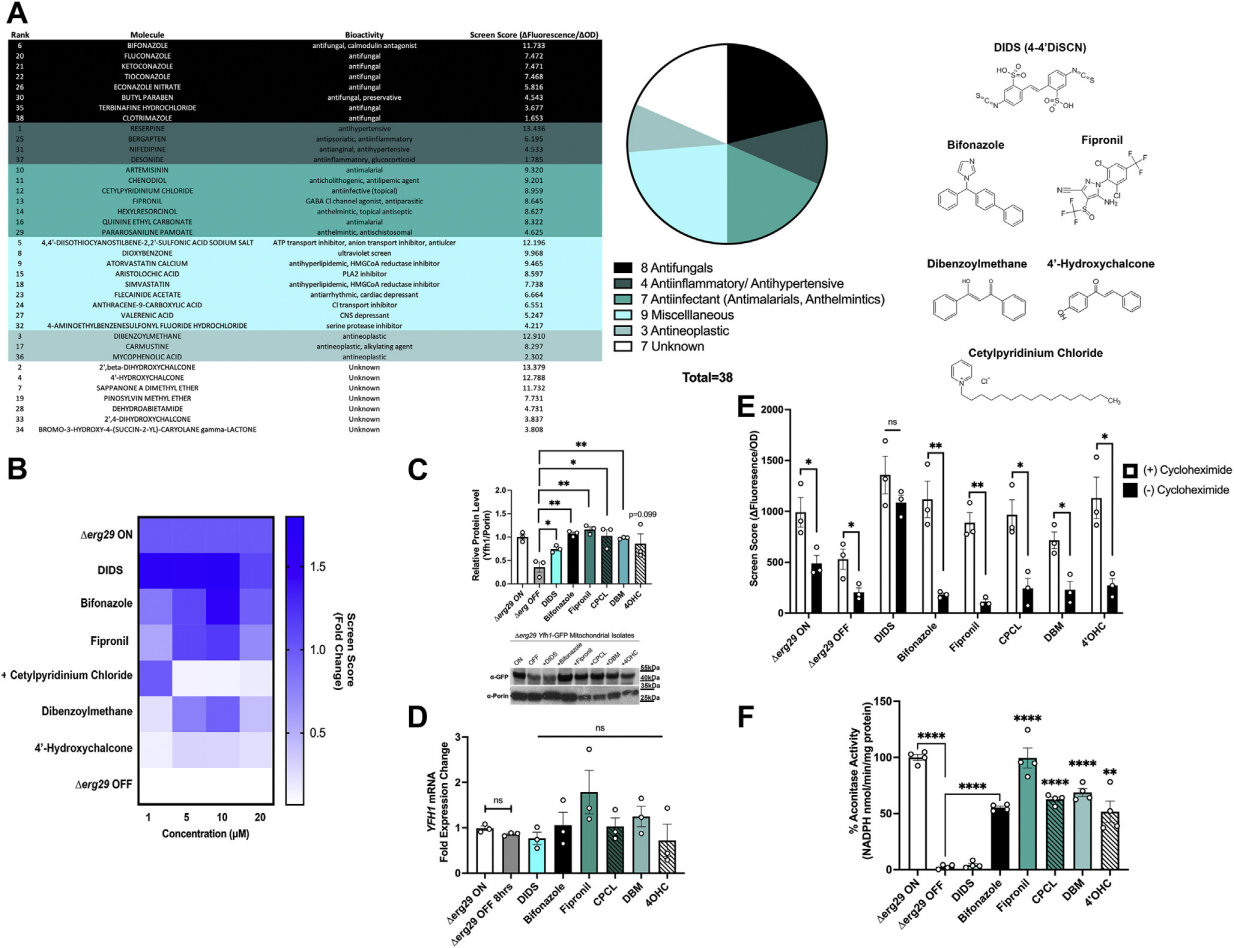
**A high throughput screen identified multiple compounds that stabilize Yfh1 levels and promote growth in the absence of Erg29.***A*, a screen of 2500 FDA-approved compounds (10 μM) was performed as described in Experimental procedures using the Drug Discovery Core at the University of Utah. Thirty-eight compounds were identified as being effective in increasing Yfh1-GFP levels and rescuing growth deficiency. These compounds were pursued for further investigation and repeated in triplicate at a static concentration of 10 μM, ranked according to their score, and subsequently divided into mechanistic drug classifications. Chemical structures of the top six compounds are shown. *B*, the top six compounds were then tested in triplicate at four different concentrations (1, 5, 10, and 20 μM) and their respective screen scores plotted in comparison to Δ*erg29* ON and OFF. The data are presented as a heat map with the fold change over *Δerg29* ON normalized as 1.0. n = 3 replicate experiments. *C*, Yfh1-GFP levels were assessed from mitochondria isolated from yeast grown as in (*A*) using Western blot analysis with Porin levels determined as a mitochondrial loading control. A representative blot is shown and blots were quantified as described in Experimental procedures. A graph of n = 3 replicate experiments is shown. *D*, Δ*erg29* ON cells were grown in galactose and then shifted to glucose and incubated with 10 μM compounds as in (*A*). RNA was isolated and RT-qPCR for *YFH1* and *ACT* performed as described in Experimental procedures. The data were normalized to 1.0 for cells grown in galactose. Error bars represent SEM (n = 3 replicate experiments with 3–4 technical replicates/sample in each experiment). *E*, cells as in (*A*) were grown ± compounds and ± 100 μM cycloheximide for 8 h and growth (absorbance) and Yfh1-GFP levels measured by fluorescence as described in the screen in Experimental procedures. (n = 3 replicate experiments). *F*, aconitase activity was measured from cells as in (*C*). Error bars represent SEM (n = 4 replicate experiments). FDA, Food and Drug Administration; RT-qPCR, reverse transcription quantitative PCR.

Current therapies for FRDA have shown modest effects *in vivo* and there is a great need for better therapies for this lethal disorder. Studies have suggested that iron chelation, antioxidants, or increased autophagy may improve mitochondrial function in either the absence of Yfh1 (54, 55) or when Fxn levels are reduced in FRDA (56, 57). Our data suggest that increasing or stabilizing Fxn protein levels is an extremely attractive approach to treat the progression of FRDA. We used our unique *ERG29 YFH1-GFP* shut off yeast strain to identify compounds from a 2500 FDA-approved library that increased Yfh1-GFP levels in the absence of Erg29. *Δerg29pGAL1ERG29 YFH1-GFP* cells were grown in galactose (log phase) and then shifted to glucose to turn off *ERG29* expression in the presence or absence of 10 μM compounds. We measured the levels of Yfh1-GFP fluorescence and cell proliferation (absorbance) to determine compound efficacy. Fluorescence “noise” in *ERG29* ON and OFF cells was determined prior to compound analysis to ensure a significant window was available for detecting changes in GFP signal (Fig. S4, *A* and *C*). We identified 38 compounds that showed a >1.5-fold increase in Yfh1-GFP fluorescence/absorbance. The compounds were grouped according to their predicted mechanism of action (Fig. 4*A*). Compounds that showed autofluorescence (*e.g.*, reserpine) were eliminated from further analysis. The optimum concentration (1–20 μM) for top compounds from each group was determined (Figs. S4*B* and 4*B*). We note that the concentration curve heat map for the top compounds did not consistently rank the compounds; however, all compounds showed increases in Yfh1-GFP as shown by Western blot analysis quantification (Fig. 4*C*). Increases in Yfh1-GFP could be the results of increased transcription, increased mRNA stability, increased translation, or increased protein stability. To determine if compounds altered *YFH1* transcripts, we performed RT-qPCR on cells grown in the presence or absence of the top six compounds. *YFH1* expression was not significantly increased in cells treated with compounds (Fig. 4*D*). To test if the compounds affected *YFH1-GFP* translation or protein stability, we tested compound efficacy in the presence or absence of cycloheximide. Most compounds showed an inhibition in compound-mediated increases in Yfh1-GFP in the presence of cycloheximide (Fig. 4*E*) supporting that these compounds may be increasing *YFH1-GFP* translation. In contrast, incubation with cycloheximide did not prohibit the 4,4-diisothiocyanostilbene-2,2-sulfonic acid (DIDS)–mediated increases in Yfh1-GFP. This suggests that DIDS is working posttranslationally. We recognized the caveat that cycloheximide might be affecting the translation of other proteins that impact Yfh1-GFP levels. One prediction of having increased Yfh1 levels is improved ISC synthesis. Indeed, treatment with top compounds including bifonazole, fipronil, cetylpyridinium chloride (CPCL), dibenzoylmethane (DBM), and 4′ hydroxychalcone (4′-OHC) increased ISC synthesis as measured by aconitase activity (Fig. 4*F*). These results confirm that our yeast screen successfully identified compounds that increased Yfh1 levels and ISC synthesis in yeast. It was surprising that aconitase activity was not increased by DIDS. In fact, aconitase activity was reduced by 50% in *ERG29* ON cells treated with DIDS, suggesting that DIDS affects aconitase activity independent of the cells used (Fig. S4*B*). We do not know the mechanism of reduced aconitase activity due to DIDS.

### Compounds show increased levels of Fxn in doxorubicin-mediated cardiomyocyte toxicity and in iron-loaded FRDA fibroblasts

**Figure 5:**
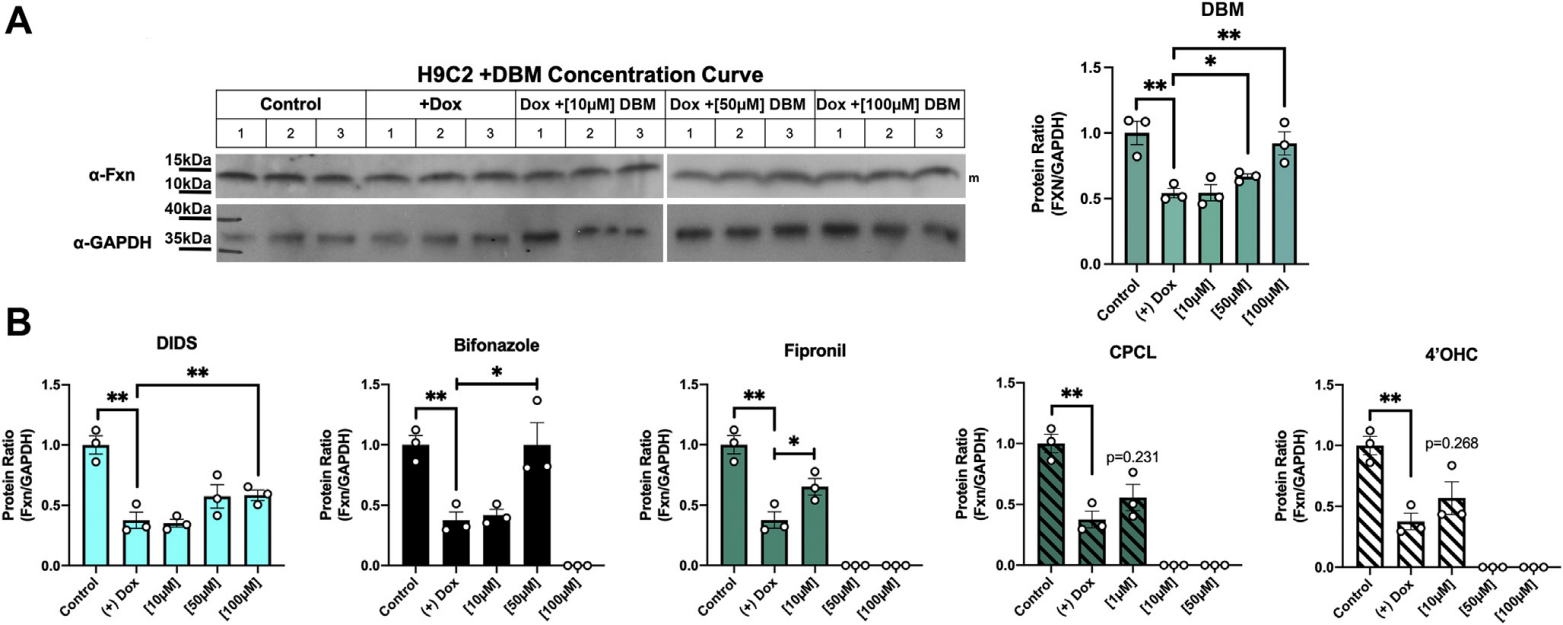
**Compounds identified from the screen show efficacy in improving Fxn levels in doxorubicin-treated cardiomyocytes.***A*, H9C2 rat cardiomyocytes were cultured in the presence of doxorubicin and 10 to 100 μM DBM for 48 h and Fxn and GAPDH levels determined by Western blot. A representative blot is shown. Quantification of three biologic replicate experiments is shown. *B*, quantification of changes in response to other top compounds from the screen are shown (n = 3 replicate experiments). DBM, dibenzoylmethane; Fxn, frataxin.

**Figure 6:**
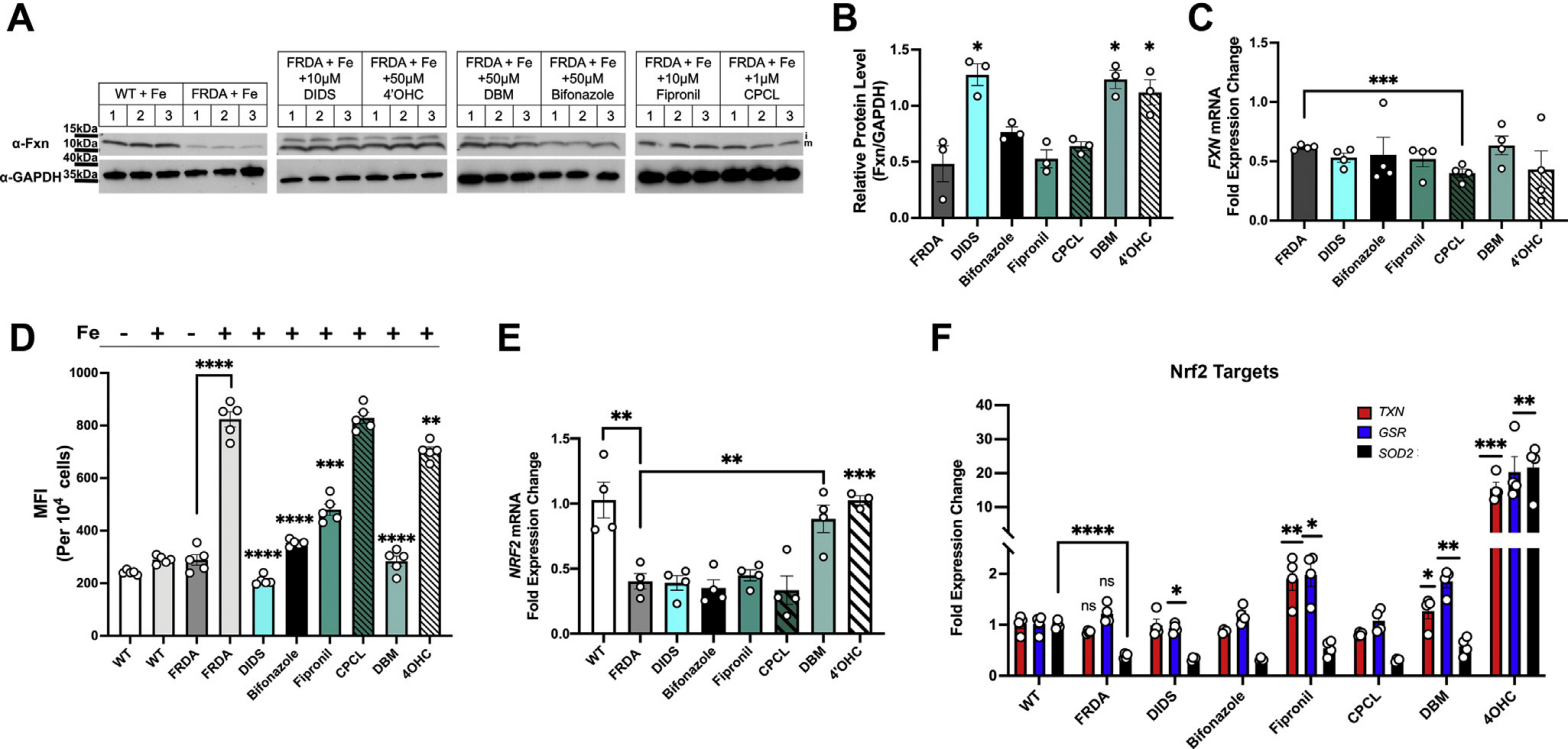
**Top compounds show increased Fxn levels in FRDA fibroblasts.***A*, WT and FRDA fibroblasts grown in the presence of 20 μM FeNTA were incubated in the absence or presence of selected compounds at the designated concentrations for 48 h and Fxn and GAPDH levels determined by Western blot. Representative blots are shown. *B*, quantification of three replicate experiments is shown normalized to WT cells. *C*, *FXN* and *ACTB* transcripts from cells as in (*A*) were determined using RT-qPCR as described in Experimental procedures. (n = 4 biologic replicate experiments with 3–4 technical replicates/experiment). *D*, mitochondrial ROS were measured using MitoSOX MFI in FRDA cells treated as in (*A*). n = 5 biologic replicates are graphed. *E*, *NRF2* and *ACTB* expression was determined from cells as in (*A*) (n = 4 biologic replicate experiments with 3–4 technical replicates/experiment). *F*, transcript levels for three transcriptional targets of Nrf2 (*TXN, GSR,* and *SOD2*) were determined from cells as in (*A*). Error bars for represent SEM (n = 3–4 biologic replicate experiments with 3–4 technical replicates/experiment). FeNTA, Fe-nitrotriacetic acid; FRDA, Friedreich’s ataxia; Fxn, frataxin; MFI,mean fluorescence intensity; ROS, reactive oxygen species; RT-qPCR, reverse transcription quantitative PCR.

To establish if any of the compounds identified showed efficacy in mammalian cells, we utilized two models, the doxorubicin cardiomyocyte model of reduced Fxn levels (23) and FRDA patient fibroblasts. The rat cardiomyocyte cell line H9C2 was grown in the presence or absence of doxorubicin and different concentrations of the top compound DBM and Fxn levels determined by Western blot. Fxn levels in doxorubicin-treated cells showed a concentration-dependent increase in the presence of DBM (Fig. 5*A*). Fxn levels were also increased with DIDS, bifonazole, and fipronil, although higher levels of bifonazole and fipronil were toxic (Fig. 5*B*). In contrast, neither 4′-OHC nor CPCL showed efficacy in increasing Fxn levels in doxorubicin-treated cardiomyocytes. In FRDA cells DIDS, DBM, and 4′-OHC showed increased Fxn levels, while bifonazole showed a trend toward increased Fxn levels, although not to significance (Fig. 6, *A* and *B*). DBM and DIDS showed efficacy in both models of decreased Fxn. We speculate that the differences in effectiveness of compounds between cardiomyocytes and FRDA fibroblasts may simply reflect cellular differences in the efficiency of compound uptake. We note that increased Fxn levels in FRDA cells were not the result of increased *FXN* expression (Fig. 6*C*) similar to that seen in the yeast system used to original screen compounds. This leaves open the question of the mechanism(s) of action of these compounds in mammalian cells. Most effective compounds showed reduced levels of mitochondrial ROS, although CPCL did not decrease mitochondrial ROS and 4′-OHC was not as effective (Fig. 6*D*). These results suggest that either compound-induced increases in Fxn itself reduces mitochondrial ROS or compounds may work by increasing antioxidants that then improve Fxn translation or protect Fxn from degradation.

Previous studies have shown that the expression of the antioxidant master regulatory transcription factor nuclear factor erythroid 2–related factor 2 (Nrf2) is diminished in FRDA (58, 59). We hypothesized that some compounds may be working through Nrf2. We confirmed that FRDA cells showed reduced *NRF2* expression, which was increased by two compounds, DBM and 4′-OHC (Fig. 6*E*). We next tested to see if the downstream activation of known transcriptional targets of Nrf2 were affected by the addition of compounds. DBM and 4′-OHC showed efficacy in improving antioxidant target transcripts including *TXN* and *GSR* but no increase in *SOD2*, whereas, 4′-OHC increased expression of all three Nrf2 targets (Fig. 6*F*). These data support that DBM, fipronil, and 4’-OHC may be working by increasing antioxidants that may protect Fxn from modification and increased turnover in FRDA. That fipronil did not increase Nrf2 but did increase antioxidant transcripts, which suggests that it may be working through a different mechanism to increase antioxidants in FRDA cells. The mechanism of how DIDS is reducing antioxidants and improving Fxn levels remains to be elucidated. Together, our results demonstrate that using yeast to identify compounds that increase Fxn levels in mammalian cell culture is a viable approach to discovering novel potential therapies for the treatment of FRDA.

## Discussion

In this study, we demonstrate that loss of Erg29 in yeast results in Lon protease Pim1-mediated degradation of yeast Fxn homolog Yfh1, while other ISC synthesis proteins Nfs1 and Isu1 levels are not reduced. We also show that Yfh1 can be protected from degradation by prohibiting mitochondrial iron accumulation through the overexpression of the mitochondrial iron exporter Mmt1. These results support the hypothesis that Fxn protein levels are sensitive to mitochondrial iron accumulation and subsequent mitochondrial ROS–mediated modification that increases Fxn turnover. The literature suggests that a decrease in Fxn due to ROS is not restricted to yeast or sterol metabolism but occurs in mammalian cells in response to drugs or conditions that affect mitochondrial ROS. Cardiac toxicity resulting from doxorubicin treatment limits the use of this drug in cancer therapy. It is well known that the toxicity of these types of drugs is iron related, and iron chelation can reduce cardiac toxicity (24, 60, 61). Recent studies demonstrated that doxorubicin decreased Fxn levels and that overexpression of Fxn or decreased mitochondrial iron through overexpression of the putative mitochondrial iron exporter Abcb8 preserved Fxn (23, 24, 61). We note that primary cardiomyocytes show increased doxorubicin toxicity compared to H9C2 cells (62, 63) making it critical to keep this in mind along with doxorubicin concentration utilized when evaluating compound efficacy for potential future studies in primary induced pluripotent stem cell–derived cardiomyocytes. In our current study, we demonstrate that mammalian Fxn shows similar sensitivity to iron-mediated mitochondrial ROS that drives mitochondrial Lon1 protease-mediated Fxn degradation, while other ISC proteins were not reduced under similar conditions. We show this in two mammalian model systems, doxorubicin-mediated toxicity in cardiomyocytes and FRDA fibroblasts grown in elevated levels of media iron. Previous studies have suggested that ISC proteins are potential targets of the Lon protease (64), but to our knowledge, this is first confirmation that Lon protease is involved in mature Fxn turnover. We recognize that inhibition of any protease system can have pleiotropic effects. That we see changes in Fxn levels and no other ISC proteins in the *ERG29* OFF or FeNTA grown FRDA cells and that deletion of *PIM1* or use of CDDO-Me protects Fxn from increased degradation strongly supports a role for the Lon protease in Fxn turnover. This observation is in contrast with the previous report that Lon was not involved in mature Fxn turnover (37). Perhaps one explanation for the differences in these two studies is that our studies utilized a *Δpim1* strain, which has no Lon protease activity, to measure Yfh1 turnover. Further, we also utilized a compound that has been shown to inhibit Lon activity and that overexpressing only Lon1 was sufficient to rescue the effects of CDDO-Me treatment, while the studies of Nabhan *et al.* utilized siRNA leaving open the possibility of remaining Lon protease activity in those studies. Another notable difference in our studies and those previously reported is that our model conditions show increased mitochondrial ROS in both yeast and mammalian cell culture conditions. That mitochondrial ROS are increased like that seen in FRDA may provide the necessary environment to modify Fxn, making it a target of the Lon protease. Previous *in vitro* studies have shown that a conserved tryptophan residue Trp155 in human Fxn is a hotspot for oxidative chemical modification (65). Studies on how Fxn might be modified in our conditions and what residues are modified are a future direction of the lab.

We speculate that when Fxn levels drop below 50% (no phenotypes are associated with a single allele mutation) (8, 9), “free” iron begins accumulating in the mitochondria giving rise to increase mitochondrial ROS and Fxn degradation without the ability to compensate by synthesizing sufficient ISCs. Our results suggest that Fxn acts like a gatekeeper involved in both ISC synthesis as well as sensing mitochondrial ROS inducing “free” iron pools, making it an “Achilles heel” in ISC synthesis and mitochondrial homeostasis. In FRDA, Fxn levels are already at a critically low level, so the discovery that this level can be further reduced due to increased mitochondrial iron and ROS may provide additional guidance on therapeutic interventions to delay disease progression. The use of iron chelators has been suggested for FRDA and studies in model systems have shown some promise (for review see (66)); however, some studies suggest that when ISC synthesis is insufficient, limiting mitochondrial iron by chelation can be detrimental by further limiting ISC synthesis (67). We hypothesize that these contrasting results may be ascribed to “timing” of iron chelation therapy. That is, once Fxn levels and ISC synthesis drop too low, biology selects to increase mitochondrial iron levels as a response to try to improve ISC synthesis. However, if Fxn levels are too low to efficiently make ISC, reducing mitochondrial iron may have deleterious effects by limiting iron for low level ISC synthesis that occurs in FRDA. This suggests that combination therapies that improve Fxn levels and ISC synthesis would be the most effective way to prevent mitochondrial iron accumulation.

The findings of our studies have implications beyond understanding the posttranslational regulation of Fxn. While the role(s) of Fxn remain to be fully elucidated, several reports describe improved consequences to increased levels of Fxn under conditions of increased mitochondrial ROS. (1) Overexpression of *FXN* in transgenic flies suppressed the toxic effects of iron, H_2_O_2_, and paraquat on lifespan (68). (2) Purified recombinant cell-permeable Fxn (PEP1-Fxn) protected neuronal cells from oxidant-mediated cell death (69). (3) The effects of the Parkinson's disease inducing neurotoxin 1-methyl-4-phenyl-1,2,3,6-tetrahydropyridine, which targets complex I in the electron transport chain can be suppressed by providing recombinant Tat-Fxn (70). These results underscore that mitochondria are highly susceptible to iron-mediated toxicity and suggest that this toxicity may target ISC formation. We speculate that other compounds/conditions that result in mitochondrial toxicity, through generation of ROS, might also attack ISC synthesis and be suppressed by either removal of mitochondrial iron or increased levels of Fxn. It is important to take into consideration that there may be consequences to “overexpression” of Fxn. Indeed, studies have suggested that if *FXN* is highly overexpressed there can be significant deleterious consequences (71, 72). Our studies and compounds discovered recover Fxn levels but not above that seen in untreated WT cells. We report that Fxn levels are increased in response to higher cellular iron levels, but we did not observe any deleterious consequences to this increase in Fxn. We further show that *FXN* transcripts are increased in response to increased cellular iron. This suggests that one role of Fxn is to protect cells/mitochondria from the consequences of elevated iron, such as when making more ISCs. To our knowledge, the generation of more ISCs is not toxic to cells, and we hypothesize that this may be a way to store iron and provide a level of protection from “free” iron toxicity.

**Figure 7:**
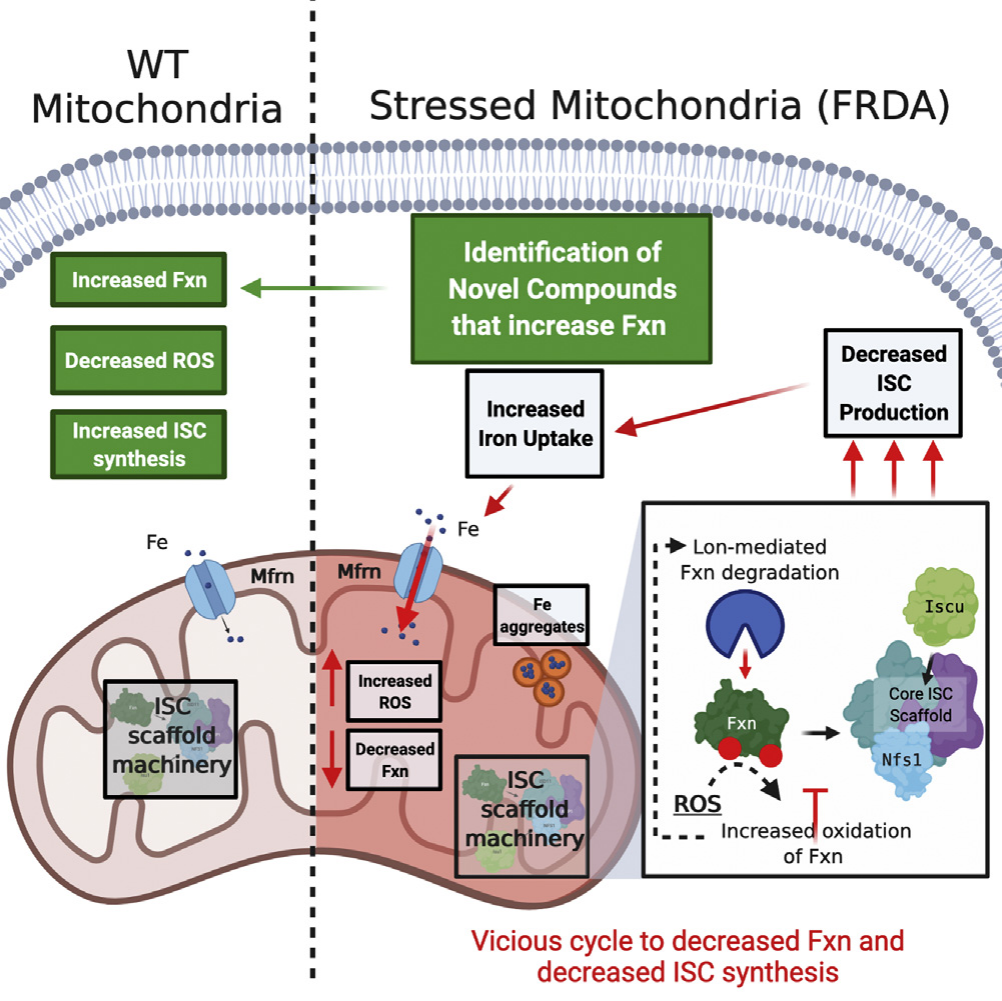
**Graphic abstract of mitochondrial stress and Fxn turnover in FRDA.** Under normal conditions WT mitochondria have an intact ISC machinery and iron import into the mitochondrial occurs at a normal rate. In FRDA, with diminished Fxn or when ISC synthesis is diminished, mitochondrial iron import is increased without improvements in ISC synthesis. This causes iron accumulation in the mitochondrial, increased mitochondrial ROS, and decreased Fxn protein levels. We speculated that Fxn is oxidized under these conditions making it susceptible to increased proteolytic degradation in the mitochondria by the Lon protease. We identified novel compounds that increased Fxn levels in FRDA cells that resulted in decreased ROS and increased ISC synthesis. We speculate that these compounds, in combination with other FRDA therapeutics, would delay disease progression. FRDA, Friedreich’s ataxia; Fxn, frataxin; ISC, Fe–S cluster; ROS, reactive oxygen species.

We utilized our novel yeast strain Δ*erg29pGAL1ERG29YFH1-GFP* to screen for compounds that prevent decreases in yeast Fxn and those compounds showed efficacy in improving Fxn levels in mammalian models including FRDA cells (Fig. 7). We successfully identified compounds that did not affect *FXN* transcripts but rather affected mature Fxn protein levels. That we did not identify compounds that increased *YFH1/FXN* transcription may simply reflect the group of top compounds we chose to analyze more extensively. It is also possible that the compound library (2500 FDA approved compounds) did not contain compounds that would be effective in moderating yeast *FXN* transcription or that increased yeast *FXN* transcription might be limited in our model. We discovered compounds that increased antioxidant gene expression but we also identified compounds, bifonazole and DIDS, that appear to modulate Fxn levels through different mechanisms. Understanding how these compounds are working and discovering multiple pathways to increase Fxn levels may provide a multipronged approach to effective therapies for FRDA. Indeed, a recent study suggests that buffering the oxidative stress associated with FRDA using gold quantum cluster therapy reduced ROS, decreased autophagy, and increased Fxn protein expression providing a novel therapeutic strategy to delay disease progression in FRDA (73). This study along with others and our work reported here suggests that there may be novel therapeutic approaches to reduce disease progression in FRDA.

## Experimental procedures

### Yeast, plasmids, and growth medium

Genotypes of strains employed in this study are listed in Table 1. The WT strains employed for these experiments were from the W303 background. Most deletion strains were created by either PCR amplifying the KanMX cassette from the homozygous diploid deletion collection (Research Genetics) or fusion PCR (74). Cells were grown in culture medium (0.67% yeast nitrogen base, 0.12% dropout amino acid mixture, ± 2% dextrose, or 4% galactose).

**Table 1:** Yeast strains

Strain	Genotype	Note
DY1457	*ura3-52, leu2-3,112, trp1-1, his3-11, 15, ade6, can1- 100(oc)*	(75)
DY1457 *YFH1-GFP*	*ura3-52, leu2-3,112, trp1-1, 15, ade6, can1- 100(oc), YFH1-GFP::HIS3*	This study
*Δerg29 pβ-estradiol (pGEV)GAL1ERG29 YFH1-GFP*	*ura3-52, leu2-3,112, trp1-1, his3-11, 15, ade6, can1- 100(oc) Δerg29::KanMX4 pβ-estradiol (pGEV)GAL1ERG29- 6XHIS YFH1-GFP::HIS3*	This study
*Δpim1 YFH1-GFP*	*ura3-52,112, trp1-1, 15, ade6, can1- 100(oc), YFH1-GFP::HIS3 Δpim1::LEU2*	This study
*Δerg29 pβ-estradiol (pGEV)GAL1ERG29 Δpim1 YFH1-GFP*	*ura3-52,112, trp1-1, his3-11, 15, ade6, can1- 100(oc) Δerg29::KanMX4 pβ-estradiol (pGEV)GAL1ERG29-6XHIS YFH1-GFP::HIS3 Δpim1::LEU2*	This study
*Δerg29pβ-estradiol (pGEV)GAL1ERG29 YFH1-GFP*	*ura3-52, leu2-3,112, trp1-1, his3-11, 15, ade6, can1- 100(oc) Δerg29::KanMX4 pβestradiol-GAL1ERG29- 6XHIS YFH1::HIS3*	This study
*Δerg29pβ-estradiol (pGEV)GAL1ERG29 NFS1-GFP*	*ura3-52, leu2-3,112, trp1-1, his3-11, 15, ade6, can1- 100(oc) Δerg29::KanMX4 pβestradiol-GAL1ERG29- 6XHIS NFS1-GFP::HIS3*	This study
*Δerg29pβ-estradiol (pGEV)GAL1ERG29 YFH1-GFP pMMT1*	*ura3-52, leu2-3,112, trp1-1, his3-11, 15, ade6, can1- 100(oc) Δerg29::KanMX4 pβestradiol-GAL1ERG29- 6XHIS YFH1-GFP::HIS3 pMMT1*	This study

### Mammalian cell culture

Fibroblasts (WT or FRDA Coriell GM03816) or rat H9C2 cardiomyocytes were grown in Dulbecco’s minimal essential medium with 10 to 15% fetal bovine serum and Pen/Strep and H9C2 with an additional 2 mM glutamine.

### Generating chromosomally integrated *Yfh1-GFP* yeast strains

In order to avoid artifacts of overexpression, a GFP tag was chromosomally integrated in frame into the C terminus of the coding sequence of *YFH1*. The linear DNA sequence was amplified from the WT strain using primers as described in Table 2 also contained a His selectable marker used to transform both the WT and *Δerg29pERG29* strains followed by transformation with a plasmid containing a *GAL1*-regulated *ERG29*. The *Δerg29pERG29pGAL1ERG29* strain was then placed on 5′-fluoroorotic acid to allow the loss of the *pERG29* plasmid. The chromosomal integration and selection of clones was confirmed *via* PCR as well as Western blotting.

**Table 2:** Yeast plasmids

Plasmid	Note
p*GAL1ERG29*	*TRP1*
p*MMT1*	*LEU2*

### Cycloheximide pulse chase

Cells were incubated with cycloheximide at a final concentration of 35 μg/ml and harvested at 0 h, 2 h, 4 h, 6 h, and 8 h time points. At each time point the *A*_600_ was measured to ensure that the cell count was normalized for each collection. Mitochondria were isolated from each sample as previously described. The half-life of each protein was calculated using nonlinear regression one phase decay.

### High throughput screen

*Δerg29pGAL1ERG29YFH1-GFP* cells were grown overnight in the presence of 4% galactose. The media were made iron deficient by addition of 80 μm bathophenanthroline sulfonate with ferrous sulfate (5 μM) added back. The cells were then harvested and washed once with water before being resuspended in galactose or glucose-containing media at an *A*_600_ of 0.01, then seeded onto a 96-well plate (100 μl). Drugs were added at a static concentration of 10 μM using a Tecan Evo 96 dispenser, utilizing the Spectrum Library (2560 compounds) provided by MicroSource Discovery Systems. Plates were incubated for 24 h, shaking at 30 °C. *A*_600_ values and fluorescence were measured at 0 h and 24 h using a Biotek Synergy plate reader. The fluorescence detection was set to the excitation/emission spectra of 380 nm/450 nm. A Z score of 0.527 was determined using *Δerg29* ON (galactose) and *Δerg29* OFF (glucose) as positive and negative controls, respectively. The score for the screen was then calculated using:

$$\Delta \left(\frac{Fluorescence}{OD_{600}+1}\right)$$

The top 42 candidates were selected for a follow-up screen with an n = 3 for each drug and then scored accordingly. After eliminating false positive compounds with intrinsic fluorescence, a concentration curve (1 μM, 5 μM, 10 μM, and 20 μM) was established using the top 18 highest scoring compounds to determine the most efficacious concentration.

### Crosslinking beads

Crosslinked beads were created using magnetic Dynabead M-280 Sheep anti-rabbit immunoglobulin (Ig) G beads that were covalently linked to rabbit monoclonal anti-GFP antibody (Abcam; ab6556). Briefly, antibody was added to beads at a concentration of 0.2 μg per 10^7^ beads. The antibody was then covalently crosslinked using 20 mM dimethyl pimelimidate in 0.2 M triethanolamine, pH 8.2, and unbound Ig was removed using 0.1 M Na citrate buffer pH 2.0.

### MitoSOX

*Δerg29pGAL1ERG29* cells were grown in galactose overnight and then shifted to glucose to shut off *ERG29* expression for time. MitoSOX was added at a final concentration of 0.625 μM, cells incubated for 45 min at 30 °C in a roller drum, washed twice with 1 ml of ice-cold PBS, and cells resuspended in 500 μl of PBS. MitoSOX fluorescence was detected using a BD FACSCanto running FACSDiva version 8.0 software from BD Biosciences as described previously (26). For mammalian cells, cells were incubated in 5 μM MitoSOX in Hank’s balanced salts solution for 10 min at 37 °C, washed twice, and resuspended in 1 ml buffer prior to fluorescence detection.

### Sterol extractions

Sterols were extracted and detected using GC-MS as previously described (26).

### RT-qPCR

Total RNA was isolated using Agilent Technologies mini kit. The SuperScript III kit from ThermoFisher Invitrogen was used to synthesize first-strand complementary DNA from total RNA. Power SYBR Green Master mix (Life Technologies) was used on a Realplex2 thermal cycler (Eppendorf). β-Actin was used as a control housekeeping gene. The ΔΔCt method was used to compare the variation of transcripts among samples. Specificity and efficiency were checked before using this method. RT-qPCR primers used are as described in Table 3.

**Table 3:** Primers

Primer	Sequence (5’ – 3′)
Δ*pim1* FWD	GTTTAGTTGTTTTTTCTTTTGGTTTTCGAGGTGCTTGAACGAAAAGATTTGCAAATAGAGCatcttgaccgcagtt
Δ*pim1* REV	ATGTTTAAATATTTACAGAATGTTTAAACAGGTATTTAATCCATTTAGATGAAAAGTTAGTgtgtcgtttctattatgaatttc
*YFH1-GFP* FWD	CATTGTCCGGGCCTAACAGA
*YFH1-GFP* REV	CATACATACACACACGGTAC
Δ*pim1* check FWD	CCTTAGGATTCGAGAACTATGCAGAGGTGT
Δ*pim1* check REV	CCTGGCAAAACGACGATCTTCTTAGGG
Δ*pim1* check (-) REV	TACGTCACGCACGGTCAAGG
*YFH1* FWD RT-qPCR	AGCGGTCTCTCGCAAGTTTA
*YFH1* REV RT-qPCR	GCTGGACAAAATCTGGCACG
*FXN* FWD RT-qPCR	GCCTCAACCAGATTTGGAATGTC
*FXN* REV RT-qPCR	AGTCCAGCGTTTCCTCTGCTAG
*NRF2* FWD RT-qPCR	ACACGGTCCACAGCTCATC
*NRF2* REV RT-qPCR	TGTCAATCAAATCCATGTCCTG
*TXR* FWD RT-qPCR	GTAGTTGACTTCTCAGCCACGTG
*TXR* REV RT-qPCR	CTGACAGTCATCCACATCTACTTC
*GSR* FWD RT-qPCR	TATGTGAGCCGCCTGAATGCCA
*GSR* REV RT-qPCR	CACTGACCTCTATTGTGGGCTTG
*SOD2* FWD RT-qPCR	CTGGACAAACCTCAGCCCTAAC
*SOD2* REV RT-qPCR	AACCTGAGCCTTGGACACCAAC
*ACTB* FWD RT-qPCR	CAGCCTTCCTTCCTGGGTATG
*ACTB* REV RT-qPCR	AGGGTGTAAAACGCAGCTCA

Abbreviations: FWD, forward; REV, reverse.

### Other procedures and reagents

Reagents used in this study are described in Table 4. Protein determinations were performed using the bicinchoninic acid assay (Pierce) detection reagent from Thermo Fisher Scientific. Compounds were purchased from Microsource Discovery Systems Inc. Lon1 inhibitor CDDO-Me was obtained from Sigma. Proteins were analyzed by 4 to 20% or 15% SDS-PAGE Tris/glycine followed by Western blot analysis using Western Lightning (PerkinElmer Life Sciences). Antisera used for probing Western blots included anti-GFP (Sigma; 1:1000), antiyeast Nfs1 (1:1000), antiyeast Isu1 (1:2000), rabbit antimammalian Nfs1 (ThermoFisher; 1:1000), antimammalian Iscu (ThermoFisher; 1:1000), antiyeast Porin (ThermoFisher; 1:1000), anti-Fxn (Abcam; 1:1000), anti-GAPDH (Abcam; 1:5000), anti-VDAC (ThermoFisher; 1:1000), and antiaconitase (Sigma; 1:5000). Secondary antibodies were either peroxidase-conjugated goat anti-rabbit IgG or peroxidase-conjugated goat antimouse IgG (Jackson ImmunoResearch Laboratories; 1:5000). Western blots were quantified using NIH Fiji software.

**Table 4:** Key Resources Table

Reagent type (species) or resource	Designation	Source or reference	Identifiers	Additional information
Cell Line (*Homo sapiens*)	WT fibroblasts	ATCC		
Cell Line (*Homo sapiens*)	FRDA fibroblasts	Coriell	*GM03816*	
Cell Line (*Ratticus norvegicus*)	H9C2 cardiomyocytes	Franklin Lab		
Chemical compound, drug	Cycloheximide	Sigma	*CAS# 66-81-9*	125 μM
Chemical compound, drug	BPS (bathophenanthroline sulfonate)	Sigma	*CAS#52746-49-3*	80 μM
Chemical compound, drug	Ferrous sulfate	Sigma	*CAS#7782-63-0*	5 μM
Other	Dynabead M-280 Sheep anti-rabbit IgG	ThermoFisher	*CAT#11203D*	
Chemical compound, drug	Dimethyl pimelimidate	ThermoFisher	*CAT#21667*	20 mM
Chemical compound, drug	Triethanolamine	Sigma	*CAS# 102-71-6*	0.2 M
Chemical compound, drug	MitoSOX	ThermoFisher	*CAT#M36008*	5 μM
Chemical compound, drug	CDDO-Me	Sigma	*CAS#218600-53-4*	2 μM
Chemical compound, drug	Screen compound library	Microsource Discovery Systems		
Commercial assay or kit	Agilent Tech. Mini RNA kit		*Prod #5185-6000*	
Antibody	Rabbit anti-GFP	Abcam	*Ab6556*	
Antibody	Mouse anti-GFP	Sigma	*SKU#11814460001*	.2 ug/10^7^ beads
Antibody	Rabbit anti-Nfs1 (yeast)			1:1000
Antibody	Rabbit anti-Nfs1 (mammalian)	ThermoFisher	*Ref# PA5-76984*	1:1000
Antibody	Rabbit antiyeast Isu1			1:5000
Antibody	Rabbit antimammalian Iscu	ThermoFisher	*CAT# PA5-70181*	1:5000
Antibody	Mouse antiyeast Porin	ThermoFisher	*CAT#459500*	1:5000
Antibody	Rabbit anti-Fxn	Abcam	*Ab175402*	1:1000
Antibody	Rabbit anti-GAPDH	Abcam	*Ab9485-100*	1:1000
Antibody	Rabbit antiaconitase 2	ThermoFisher	*CAT# PA5-19269*	1:5000
Antibody	Rabbit anti-VDAC	ThermoFisher	*CAT#PA1-954A*	1:10,000
Antibody	Secondary peroxidase conjugated antibodies	Jackson Immuno Research		1:1000
Commercial assay or kit	SuperSignal West Femto	ThermoFisher	*CAT#34095*	1:5000
Commercial assay or kit	Western lighting plus ECL	PerkinElmer	*NEL104001EA*	
Software, algorithm	NIH Fiji	Imagej.net/fiji		
Software, algorithm	GraphPad Prism Version 9.0.1	www.graphpad.com		

### Statistics

All experiments were performed a minimum of n = 3 times with many individual experiments including multiple biologic replicates as noted in the figure legends. Statistical analyses were performed using Student's *t* test with significance set at *p* ≤ 0.05, where *p* ≤ 0.05 is shown as ∗, *p* ≤ 0.01 = ∗∗, *p* ≤ 0.001 as ∗∗∗, and *p* ≤0.0001 as ∗∗∗∗.

## Data availability

All data are contained within the article.

## Supporting information

This article contains supporting information.

## Conflict of interest

The authors declare that they have no conflicts of interest with the contents of this article.

## Figures and Tables

**Supplemental Figure 2:**
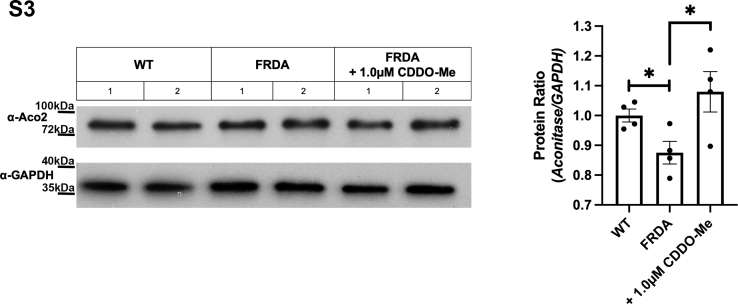


**Supplemental Figure 4:**