# Supplementary material for: Posttranslational regulation of mitochondrial frataxin and identification of compounds that increase frataxin levels in Friedreich’s ataxia
Source: J Biol Chem. 2022 Apr 25;298(6):101982. doi: 10.1016/j.jbc.2022.101982 (PMC9127368; doi:10.1016/j.jbc.2022.101982)
Supplement: Supporting information [file mmc1.docx]

**Supporting information Figure Legends Hackett et al**

Sup Figure 1 Yfh1-GFP localizes to the mitochondria

*∆erg29pGAL1ERG29YFH1-GFP* (red) cells were grown in galactose (ON) to log phase and incubated with 5 µM Mitotracker Red at 30ºC for 30 minutes, cells washed and attached to glass coverslips coated with Concanamycin A. Coverslips were mounted onto glass slides and images captured using an Olympus BX51 Fluorescence Microscope and QuantOne Software with a 100x oil immersion lens 1.3x aperture. DIC = Differential Interference Contrast. Images were processed as .tiff files in Adobe Photoshop.

Sup Figure 3 Aconitase2 degradation is prohibited by the Lon protease inhibitor CCDO-Me

WT and FRDA fibroblasts were grown in 20 µM FeNTA for 24 hours +/- 1.0 µM CDDO-Me. Cells were harvested and Aconitase2 and GAPDH levels determined by Western blot. A representative blot is shown with two biologic replicates. Blots were quantified using Fiji Image J from 4 independent biologic replicates. Error bars represent SEM, p< 0.05.

Sup Figure 4 Compound validation and optimized concentrations for Yfh1-GFP improvement in *∆erg29pGAL1ERG29YFH1-GFP* (OFF) yeast

1. *∆erg29pGAL1ERG29YFH1-GFP* cells were grown in galactose (ON) to log phase and shifted to glucose (OFF) +/- compounds for 24 hours (three biologic replicates/ compound/concentration). Yfh1-GFP fluorescence and cell proliferation were measured as described in Experimental procedures. Fold change is shown for 17 compounds as a heat map as in figure 4. B. Aconitase activity was measured in cells as in A +/- 10 µM DIDS as described in Experimental procedures. Error bars represent SEM, p< 0.05 (n=4). C. An example Z’ factor of fluorescence data generated from the 96 well format for cells as in A grown in galactose or glucose is shown. Z’ factor describes the available signal window for an assay in terms of the total separation between *ERG29OFF* and *ERG29ON* minus the error associated with each type of control (0.579). The fluorescence data are expressed as Arbitrary Units.
